# Roadmap for the expression of canonical and extended endocannabinoid system receptors and metabolic enzymes in peripheral organs of preclinical animal models

**DOI:** 10.14814/phy2.15947

**Published:** 2024-02-26

**Authors:** J. J. Rosado‐Franco, A. L. Ellison, C. J. White, A. S. Price, C. F. Moore, R. E. Williams, L. B. Fridman, E. M. Weerts, D. W. Williams

**Affiliations:** ^1^ Department of Pharmacology and Chemical Biology Emory University School of Medicine Atlanta Georgia USA; ^2^ Department of Molecular and Comparative Pathobiology Johns Hopkins University‐School of Medicine Baltimore Maryland USA; ^3^ Department of Molecular Microbiology and Immunology Johns Hopkins University‐Bloomberg School of Public Health Baltimore Maryland USA; ^4^ Department of Psychiatry and Behavioral Sciences Johns Hopkins University Bayview Campus Baltimore Maryland USA; ^5^ Department of Neuroscience Johns Hopkins University‐School of Medicine Baltimore Maryland USA; ^6^ Division of Clinical Pharmacology Johns Hopkins University‐School of Medicine Baltimore Maryland USA

**Keywords:** cannabinoids, endocannabinoid system, mice, non‐human primate, rat, receptors

## Abstract

The endocannabinoid system is widely expressed throughout the body and is comprised of receptors, ligands, and enzymes that maintain metabolic, immune, and reproductive homeostasis. Increasing interest in the endocannabinoid system has arisen due to these physiologic roles, policy changes leading to more widespread recreational use, and the therapeutic potential of *Cannabis* and phytocannabinoids. Rodents have been the primary preclinical model of focus due to their relative low cost, short gestational period, genetic manipulation strategies, and gold‐standard behavioral tests. However, the potential for lack of clinical translation to non‐human primates and humans is high as cross‐species comparisons of the endocannabinoid system have not been evaluated. To bridge this gap in knowledge, we evaluate the relative gene expression of 14 canonical and extended endocannabinoid receptors in seven peripheral organs of C57/BL6 mice, Sprague–Dawley rats, and non‐human primate rhesus macaques. Notably, we identify species‐ and organ‐specific heterogeneity in endocannabinoid receptor distribution where there is surprisingly limited overlap among the preclinical models. Importantly, we determined there were no receptors with identical expression patterns among mice (three males and two females), rats (six females), and rhesus macaques (four males). Our findings demonstrate a critical, yet previously unappreciated, contributor to challenges of rigor and reproducibility in the cannabinoid field, which has implications in hampering progress in understanding the complexity of the endocannabinoid system and development of cannabinoid‐based therapies.

## 1 INTRODUCTION

The endocannabinoid system (ECS) has been evolutionarily conserved to preserve its importance in maintaining immune, metabolic, and reproductive homeostasis (Acharya et al., 2017; Elphick & Egertová, 2001; McPartland et al., 2006; Moreno et al., 2021). This system is present in all vertebrate animals, including rodents, non‐human primates (NHP), and humans (Elphick & Egertová, 2001; Rodríguez de Fonseca et al., 2005). The canonical ECS is comprised of two main cannabinoid receptors (coded by the *cnr1* and *cnr2* genes), endogenous lipid ligands (endocannabinoids, i.e., anandamide and 2‐arachydonoil glycerol [2‐AG]), and enzymes involved in endocannabinoid metabolism (coded by the *faah* and *naaa* genes, among others not included in this study) (Ashton, 2001; Moreno et al., 2021). There are additional extensions to the canonical ECS, termed the “extended” ECS, that are comprised of receptors with primary functions in other pathways that have accessory functions that exist upon interaction with cannabinoids (Cristino et al., 2020; Veilleux et al., 2019). Some of these receptors include peroxisome proliferator activated receptors (coded by the *ppara* and *pparg* genes, respectively), “endocannabinoid‐like” G‐protein coupled receptors (i.e., *gpr18*, *gpr55*, and *gpr119*), nociception ion channels (coded by the *trpv1* and *trpv2* genes, respectively), and transporters (i.e., *htr1a*, *adora2a*, and *adgrf1*) (Kienzl et al., 2020; Stasiulewicz et al., 2020). Though their primary functions are best characterized in other pathways, the extended ECS receptors functionally interact with endocannabinoid ligands, the phytocannabinoids present in the *Cannabis* plant, and other endogenous lipid mediators, including oleoyl‐ethanolamide (OEA), palmitoyl‐ethanolamide (PEA), and linoleoyl‐ethanolamide (LEA) (Kienzl et al., 2020; Stasiulewicz et al., 2020). Together, the canonical and extended ECS, known as the “endocannabinoidome,” consists of many receptors that can interact with multiple ligands, thus creating a complicated network of outcomes during both health and disease and not limited to the brain.

More widespread accessibility of phytocannabinoids for medicinal and recreational use, policy changes that have impacted funding priorities, and the heightened desire for plant‐based therapeutics have re‐awakened scientific interest in the ECS. As such, preclinical animal models are becoming increasingly important in identifying the health implications of phytocannabinoids and the molecular mechanisms by which the ECS can be therapeutically leveraged. However, challenges exist in the translational capacity of preclinical studies due to conflicting reports that arise because of key differences in study design, including the route of administration, formulation, dose, metabolism, animal species used, the company obtained from, sex, and fasting state (Manwell, Charchoglyan, et al., 2014; Manwell, Ford, et al., 2014; Moore et al., 2021; Moore & Weerts, 2022; Sharkey & Wiley, 2016; Wiley et al., 2007). Further, clinical translation from rodents to primates is often lost due to discrepant findings that exist among preclinical models (Matsuzaki et al., 1987; McMahon et al., 2005). Therefore, a more comprehensive understanding of the distribution of the canonical and extended ECS among preclinical animal models is necessary to increase scientific rigor and provide critical insight into the mechanisms by which phytocannabinoids elicit unexpected or seemingly contradictory findings across research groups.

To address this, we determined the relative expression of the 14 canonical and extended ECS genes (adgrf1, *adora2a, cnr1, cnr2, gpr18, gpr55, gpr119, faah, htr1a, naaa, ppara, pparg, trpv1*, and *trpv2)* in seven peripheral organs with metabolic and/or immune functions (colon, heart, kidney, liver, mesenteric lymph node [MLN], spleen, and visceral fat) in three translationally relevant preclinical animal models: C57BL/6 mice (*Mus musculus*), Sprague–Dawley rats (*Rattus norvegicus*), and rhesus macaques (*Macaca mulatta*). Of note, while our present focus was on ECS relative gene distribution in the periphery, a subsequent publication will characterize distribution across sub‐anatomic brain regions of these same animals. Further, our focus was on evaluation of ECS genes that interact with phytocannabinoids and as such we did not evaluate the “endocannabinoidome” in totality.

## 2 MATERIALS AND METHODS

### 2.1 Ethics statement

Animals and procedures in this study were approved by the Johns Hopkins University Animal Care and Use Committee. Animal handling and euthanasia were conducted as stated under the NIH Guide for the Care and Use of Laboratory Animals and USDA Animal Welfare Regulation. Rats and NHP included in this study were healthy uninfected animals serving as controls in other experiments (Moore & Weerts, 2022).

### 2.2 Animal use

#### 2.2.1 Mice

Five C57/BL6 mice (three male, two female) were included in this study. Mice were housed in ventilated racks with a 14/10‐hours light/dark cycle, with water and standard chow diet (Teklad Diet 2018; IN, USA) ad libitum. Mice were kept in their cages for 13–15 weeks before they were sedated with isoflurane and euthanized by cervical dislocation. During necropsy, colon, heart, kidney, liver, spleen, and visceral fat tissue were collected, washed with 1X PBS to remove contaminating blood, and were flash frozen with liquid nitrogen and stored at −80°C until further use. No MLN was included in this study due to the complexity of identifying them due to their small size and dissecting both the brain and the periphery at the time of necropsy. All necropsies were performed at 8 am to minimize the effect of circadian cycles on endocannabinoid receptor expression.

#### 2.2.2 Rats

Six female Sprague–Dawley rats (six female, Charles River, MA, USA) were single‐housed in wire‐topped plastic cages in temperature and humidity‐controlled facilities with a reverse light cycle (12 h, lights off from 8:00 am‐8:00 pm). Rats were provided corn‐based chow (Teklad Diet 2018; IN, USA), and water ad libitum, except when actively participating as control subjects in behavioral procedures (Moore & Weerts, 2022). Rats were 52 weeks old at the end of the study. Before necropsy, rats were sedated with isoflurane and euthanized by rapid decapitation. Upon necropsy, pancreas was the first organ to be collected and flash frozen. Afterward, colon, heart, kidney liver, MLN, and spleen were collected, washed with fresh 1X PBS, flash frozen using dry ice, and stored at −80°C until further use. No fat tissue was included in this study. All necropsies were performed at 8 am to minimize the effect of circadian cycles on endocannabinoid receptor expression.

#### 2.2.3 Non‐human primates

Four adult, male, pathogen‐free Rhesus macaques (RM) (four male, *Macaca mulatta*) were included in this study (animal identification numbers 560, 561, 562, and 563). Female macaques were not included in this study due to their importance in breeding for maintaining the colony. Macaques were pair‐house to minimize any immunologic stress caused by being single‐housed, and they were fed standard monkey chow (Teklad Diet 2018; IN, USA) (Castell et al., 2022). Macaques were 7.89, 8.76, 8.59, and 7.95 years old at time of necropsy. During necropsy, animals were sedated using ketamine and euthanized with an overdose of sodium pentobarbital, according to the American Veterinary Medical Association guidelines (2013). Phosphate buffered saline (1X) was used to perfuse organs and remove blood from organs, tissues, and MLN were taken to analyze the relative expression of the canonical, and the extended endocannabinoid receptors. No colon samples were available at the time of the study. All necropsies were performed at 8 am to minimize the effect of circadian cycles on endocannabinoid receptor expression.

### 2.3 RNA extraction and cDNA synthesis

RNA was extracted using RNeasy Mini kit (Qiagen Cat#74104, MD, USA) following manufacturer's instructions. Briefly, ~200 mg of each tissue was added to tubes containing Lysing Matrix D (MP Biomedicals Cat# #116913050‐CF, CA, USA). Tissue was homogenized using MP FastPrep®‐24 (MP Biomedicals, CA, USA). Fat tissue was centrifuged after homogenization to remove the top layer of fat as instructed by the manufacturer. Afterward, the aqueous phase was mixed with 70% ethanol (The Warner Graham Company, MD, USA) at a 1:1 ratio in a clean tube and loaded into the RNeasy columns. RNA‐free DNase (Qiagen Cat #79256, CA, USA) was added to the column to digest any DNA present in the sample, as suggested by the manufacturer. RNA concentration and quality parameters were determined using Nanodrop (ThermoFisher Scientific, MA, USA). Extracted RNA was used to synthesize cDNA using iScript cDNA Synthesis kit (Bio‐Rad Cat# #1708891, CA, USA) following manufacturer's instructions.

### 2.4 Real‐time quantitative polymerase chain reaction

Relative genetic expression was determined using real‐time quantitative polymerase chain reaction (RT‐qPCR) (CFX96™ Real‐Time System, Bio‐Rad, CA, USA) using commercially available TaqMan primers (Tables 1, 2, 3) and TaqMan™ Fast Universal PCR Master Mix (2X) no AMPERASE™ UNG (ThermoFisher Scientific, Catalog# #4367846, MA, USA). Amplification was done in 40 cycles with the following conditions (Denaturing at 95°C for 20 seconds and annealing and extending at 60°C for 20 s). Cycle threshold values were normalized using Pan Eukaryotic 18S (ThermoFisher Scientific Cat# 4333760F, MA, USA), transformed using the 2^−∆CT^ method, and graphed to represent the relative genetic expression by sample, gene group, and species.

**TABLE 1:** List of primers used to determine the relative expression of canonical and extended endocannabinoid receptors in mice (*Mus musculus*).

Gene symbol	Assay ID	Company
*adgrf1*	Mm00505409_m1	ThermoFisher Scientific
*adora2a*	Mm00802075_m1	ThermoFisher Scientific
*cnr1*	Mm01212171_s1	ThermoFisher Scientific
*cnr2*	Mm00438286_m1	ThermoFisher Scientific
*faah*	Mm00515684_m1	ThermoFisher Scientific
*gpr18*	Mm0122454_m1	ThermoFisher Scientific
*gpr55*	Mm03978245_m1	ThermoFisher Scientific
*gpr119*	Mm00731497_s1	ThermoFisher Scientific
*htr1a*	Mm00434106_s1	ThermoFisher Scientific
*naaa*	Mm01341699_m1	ThermoFisher Scientific
*ppara*	Mm00440939_m1	ThermoFisher Scientific
*pparg*	Mm00440940_m1	ThermoFisher Scientific
*trpv1*	Mm01246302_m1	ThermoFisher Scientific
*trpv2*	Mm00449223_m1	ThermoFisher Scientific

**TABLE 2:** List of primers used to determine the relative expression of canonical and extended endocannabinoid receptors in rats (*Ratus norvegicus*).

Gene symbol	Assay ID	Company
*adgrf1*	Rn01511909_m1	ThermoFisher Scientific
*adora2a*	Rn00583935_m1	ThermoFisher Scientific
*cnr1*	Rn03037213_s1	ThermoFisher Scientific
*cnr2*	Rn01637601_m1	ThermoFisher Scientific
*faah*	Rn00577086_m1	ThermoFisher Scientific
*gpr18*	Rn01493247_m1	ThermoFisher Scientific
*gpr55*	Rn03037213_s1	ThermoFisher Scientific
*gpr119*	Rn01648212_m1	ThermoFisher Scientific
*htr1a*	Rn01637601_m1	ThermoFisher Scientific
*naaa*	Rn01768319_m1	ThermoFisher Scientific
*ppara*	Rn00566193_m1	ThermoFisher Scientific
*pparg*	Rn00440945_m1	ThermoFisher Scientific
*trpv1*	Rn00583117_m1	ThermoFisher Scientific
*trpv2*	Rn00567974_m1	ThermoFisher Scientific

**TABLE 3:** List of primers used to determine the relative expression of canonical and extended endocannabinoid receptors in Rhesus macaques (*Macaca mulatta*).

Gene symbol	Assay ID	Company
*adgrf1*	Hs00228100_m1	ThermoFisher Scientific
*adora2a*	Hs00169123_m1	ThermoFisher Scientific
*cnr1*	Hs01038522_s1	ThermoFisher Scientific
*cnr2*	Hs00275635_m1	ThermoFisher Scientific
*faah*	Hs01038678_m1	ThermoFisher Scientific
*gpr18*	Hs01649814_m1	ThermoFisher Scientific
*gpr55*	Hs00271662_s1	ThermoFisher Scientific
*gpr119*	Hs00708890_s1	ThermoFisher Scientific
*htr1a*	Hs00265014_s1	ThermoFisher Scientific
*naaa*	Hs01567916_g1	ThermoFisher Scientific
*ppara*	Hs00231882_m1	ThermoFisher Scientific
*pparg*	Hs01115513_m1	ThermoFisher Scientific
*trpv1*	Hs00218912_m1	ThermoFisher Scientific
*trpv2*	Hs00275032_m1	ThermoFisher Scientific

### 2.5 Data analysis and statistics

Data were analyzed using PRISM software version 9.0 (GraphPad Software, Inc., San Diego, CA). Determination of relative gene expression was done in duplicates and represented in graphs plotting the mean ± SD. Our limit of detection (LoD) was calculated using an average of each species probing for Pan Eukaryotic 18S (ThermoFisher Scientific Cat# 4333760F, MA USA) with a cycle threshold of 35. Please note that each sample was subtracted each own 18S value and hence can appear below the LoD but its expression was detected in a Ct value below 35. Samples that did not amplify were given an arbitrary value of 39.99. Variance between the relative expression of genes between organs and by species was determined using one‐way ANOVA. Post hoc analysis was done to determine the difference between the expression of these genes when there was statistical significance determined by one‐way ANOVA.

## 3 RESULTS

### 3.1 Both canonical ECS receptors are expressed in the spleen of mice, rats and NHP

**FIGURE 1:**
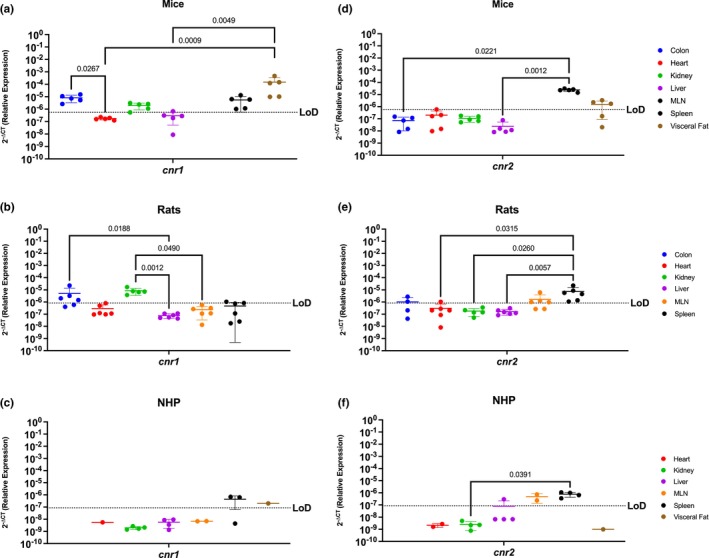
Both Canonical ECS Receptors Are Present In The Spleen Of Mice, Rats, and NHP. Relative expression of *cnr1* and *cnr2* was determined using qPCR from the colon, heart, kidney, liver, MLN, spleen, and visceral fat from mice, rats, and NHP. (a) In mice, we detected *cnr1* mRNA in the colon, kidney (4/5 mice), liver (2/5 mice), spleen, and visceral fat, having the highest levels in the latter. Significant differences were found in the levels of expression between the colon and the heart, as well as among the visceral fat and the levels of expression in the heart and the liver. (b) C*nr1* was detected in partial samples of the rat model colon (4/6 rats), heart (1/6 rats), kidney, and spleen (1/6 rats), having statistically higher levels of expression in the kidney when compared to the heart, liver, MLN, and spleen. (c) In NHP, *cnr1* mRNA was detected in the spleen (2/3 rhesus) and visceral fat at comparable levels. (d) *Cnr2* was detected only in the spleen and visceral fat (4/5 mice), at comparable levels. The spleen had statistically significantly higher levels as compared to the colon, heart, kidney, and liver. (e) *Cnr2* was detected in the colon (2/4 rats), heart (1/6 rats), MLN (4/6 rats), and spleen of rats. (f) In NHP, *cnr2* mRNA was detected in the liver (1/4 rhesus), MLN, and spleen. Detection levels were significantly higher in the spleen when compared to the heart, kidney, and liver, but with significant differences when compared to the liver (1/4 rhesus), kidney, and heart. Data are represented as the mean ± SD.

*Cnr1* is primarily expressed and studied in the context of the brain (Mackie, 2005; Pertwee, 1997). Here, we show that *cnr1* mRNA was detected in both metabolic and secondary immune organs in mice, including the colon, kidney, liver, spleen, and visceral fat (Figure 1a) Interestingly, significantly more *cnr1* was present between the colon and the heart (*p* = 0.0267), and in the visceral fat when compared to the heart (*p* = 0.0009) and the liver (*p* = 0.0049). In contrast to mice, *cnr1* expression was more restricted in rats where the highest levels occurred in kidney and the colon (Figure 1b). Indeed, *cnr1* was significantly higher in the colon when compared to the liver (*p* = 0.0188), and kidney as compared to liver (*p*‐value = 0.0012) and MLN (*p*‐value = 0.0.0490). While *cnr1* was present in colon, MLN, and spleen, it did not occur in all rats with only 4/6, 1/6, and 1/6 rats having detectable expression, respectively. *Cnr1* was least abundant in NHP, where it was limited to the spleen and the visceral fat (Figure 1c). *Cnr1* was not detectable in the liver or heart in any of the three models evaluated in this study.

*Cnr2* is primarily present in the periphery with expression in the brain occurring in the context of disease (Ellert‐Miklaszewska et al., 2007; Mackie, 2005; Pertwee, 1997). Our findings were consistent with this, where *cnr2* mRNA was detected in the spleen and in the visceral fat (4/5 mice) of mice at similar levels. Significant differences were found among the colon and the spleen (*p* = 0.0221), the liver and the spleen (*p* = 0.0012) and the liver and the visceral fat, having higher expression in secondary immune organs rather than in metabolic organs. (Figure 1d). In rats, we detected *cnr2* mRNA partially in the colon (2/4 rats), the heart (1/6 rats), MLN (4/6 rats), and the spleen (6/6 rats) (Figure 1e). Significant differences were found among the spleen and the heart (*p* = 0.0315), kidney (*p* = 0.0260), and liver (*p* = 0.0057). NHP had restricted *cnr2* mRNA, with robust levels in the MLN and spleen (Figure 1f). *Cnr2* was partially detected in the liver (1/4 rhesus) in the NHP model. Notably, *Cnr2* in the spleen was significantly more highly expressed when compared to the kidney (*p* = 0.0391).

### 3.2 Peroxisome proliferator activated receptors, *Ppara* and *Pparg*, are well conserved in all organs of mice, rats and NHP

**FIGURE 2:**
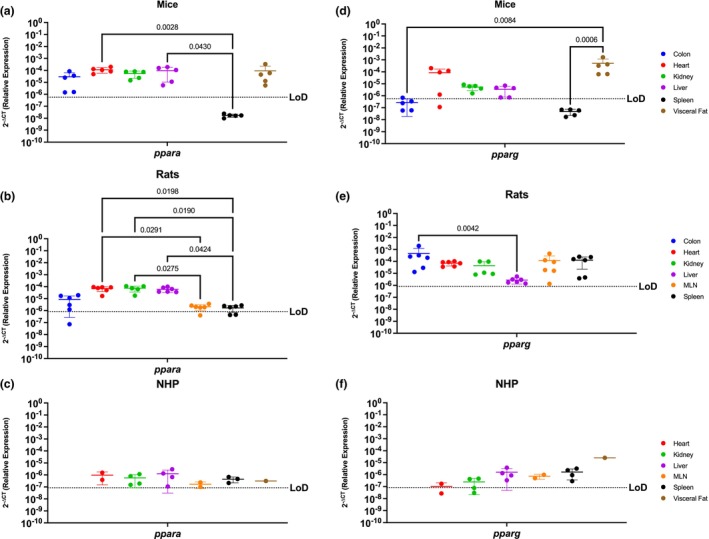
Peroxisome Proliferator Activated Receptors, *Ppara* and *Pparg*, Are Generally Well Conserved In All Organs Of Mice, Rats and NHP. Relative expression of *ppara* and *pparg* was determined using qPCR from the colon, heart, kidney, liver, MLN, spleen, and visceral fat from mice, rats, and NHP. (a) P*para* mRNA was detected in all the organs tested in mice, except for the spleen. It had significantly higher levels in the kidney when compared with the colon and the spleen. (b) In rats, *ppara* mRNA was detected in the colon (4/6 rats), heart, liver, kidney, MLN (5/6 rats), and spleen (4/6 rats). P*para* in the rat model was significantly higher in the heart, kidney, and liver, when compared to the colon, MLN, and spleen. (c) *Ppara* was detected in all organs tested in the periphery of the NHP model at comparable levels for all the evaluated organs. (d) Mouse *pparg* was similar to *ppara*, being detected in the colon (3/5 mice), heart (4/5 mice), kidney, liver (4/5 mice), and the visceral fat. Detection of this gene was higher in visceral fat when compared to the colon, kidney, liver, and spleen. (e) *Pparg* was detected in all the organs available for the rat model with no statistical significance among any organ. (f) P*parg* in the NHP model was also detected in all available organs for the NHP model, having only partial detection in the heart (1/2 rhesus) and the kidney (3/4 rhesus). Interestingly, *pparg* was significantly higher in the visceral fat when compared with all other tissues. It is worth mentioning that neither of these genes were detected in the spleen of mice, contrary to the other animal models. Data are graphed as the mean ± SD.

Peroxisome proliferator activated receptors mediate several vital functions and hence are known to be expressed almost ubiquitously (Montaigne et al., 2021; Remels et al., 2009; Rigamonti et al., 2008). Our findings corroborated this, where we determined that *ppara* mRNA was present in every evaluated organ in mice, with the notable exception of the spleen (Figure 2a). These genes were most abundantly expressed in the heart (*p* = 0.0028) and liver (*p* = 0.0430). Similar trends occurred in rats, where *ppara* mRNA was detected in all organs available, having significantly higher expression in the heart versus immune organs, such as the spleen (*p* = 0.0291) and MLN (*p* = 0.0198), in the kidney versus immune organs (*p* = 0.0275 for the MLNn and *p* = 0.0190) liver versus the spleen (*p* = 0.0424) (Figure 2b). The NHP model also showed ubiquitous *ppara*, being detected in all evaluated organs (Figure 2c).

*Pparg* mRNA followed a similar trend as *ppara* in mice, where it was detected in all organs except for the spleen and having statistically higher levels in the visceral fat when compared to the colon and the spleen (*p* = 0.0084 and *p* = 0.0006, respectively). (Figure 2d). In rats, we saw a similar scenario in which *pparg* mRNA was also expressed across all organs in rats, reaching statistically significant levels between the colon and the liver (*p* = 0.0042) (Figure 2e). In contrast, NHP had nuanced *pparg* expression where it was highly present in visceral fat, when compared to the heart (*p* = 0.0001), kidney (*p* = 0.0001) liver (*p* = 0.0001), MLN (*p* = 0.0001), and spleen (*p* = 0.0001) (Figure 2f). In sum, *ppara* and *pparg* were similarly present in all animal models, with high expression detected in all organs, with the notable exception of the spleen of mice.

### 3.3 Endocannabinoid‐like GPRs are preferentially expressed in lymphoid organs and the visceral fat

**FIGURE 3:**
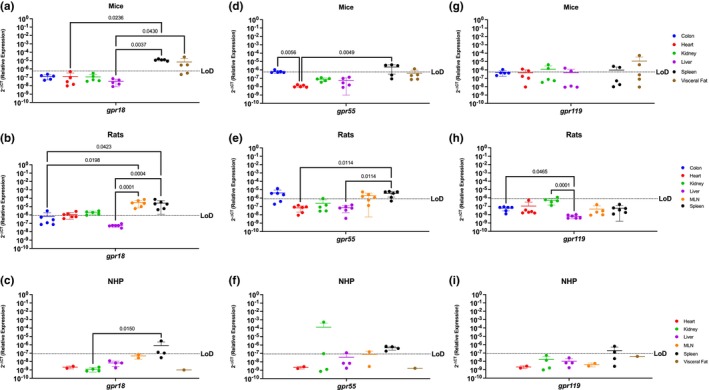
Endocannabinoid‐like GPRs Are Preferentially Expressed In Lymphoid Organs and The Visceral Fat. Relative expression of *gpr18*, *gpr55*, and *gpr119* was determined using qPCR from the colon, heart, kidney, liver, MLN, spleen, and visceral fat from mice, rats, and NHP. (a) *Gpr18* was detected in the spleen and visceral fat with significant difference between the spleen and the rest of the organs. (b) In rats, *gpr18* was highest in the secondary immune organs (MLN and spleen). (c) *Gpr18* was only detected partially in the spleen (3/4 rhesus) of the NHP model. (d) *Gpr55* was detected in the colon (1/5 mice), spleen and visceral fat (1/5 mice), having statistical significance among the spleen and all other tissues. (e) *Gpr55* expression was detected in the colon (4/6 rats), MLN (4/6 rats) and the spleen (5/6 rats). (f) In the NHP model, *gpr55* was detected in the kidney (1/4 rhesus), liver (1/4 rhesus), MLN (1/2 rhesus), and spleen (2/4 rhesus). (g) Expression of *gpr119* was detected in partially in the colon (4/5 mice), heart (2/5 mice), kidney (2/5 mice), liver (2/5 mice), spleen (2/5 mice), and visceral fat (2/5 mice). (h) *Gpr119* was only detected in the kidney (1/6 rats) with significant differences with all the other organs included in this study. (i) *Gpr119* was partially detected in the spleen (2/4 rhesus) of the NHP model. Data represents the mean ± SD.

GPRs are mostly considered to be orphan receptors until identification of their specific ligand. Some GPRs (i.e., *gpr18*, *gpr55*, and *gpr119*) are known to interact with cannabinoids and are considered to be endocannabinoid‐like GPRs (Lauckner et al., 2008; Leyva‐Illades & DeMorrow, 2013; Morales et al., 2020; Oka et al., 2007; Syed et al., 2012; Yang et al., 2018). Mice had relatively limited *gpr18* mRNA, which was present only in the colon, spleen, and visceral fat, having higher levels of expression in the spleen and visceral fat when compared to the heart (heart vs. spleen *p* = 0.0236) and liver (liver vs. spleen *p* = 0.0037 and liver vs. visceral fat *p* = 0.0430). (Figure 3a). Expression of *gpr18* mRNA was more widespread in rats than mice, having detectable levels in all organs with the exception of the liver. Highest levels of *gpr18* in rats were found between the colon and the immune organs (*p* = 0.0198 for the MLN and *p* = 0.0423 for the spleen), (Figure 3b). *Gpr18* was only partially detected in the spleen (3/4 rhesus) in the NHP model. These levels were statistically significant when compared with the kidney (*p* = 0.0150) (Figure 3c).

*Gpr55* was similar to *gpr18* in mice, being highly expressed in the spleen, colon, and visceral fat and having significantly higher levels between the spleen and heart (*p* = 0.0049) and the colon and the heart (*p* = 0.0056) (Figure 3d). *Gpr55* also primarily followed a similar expression pattern as *gpr18* in rats; however, they did not express *gpr55* in the heart or liver (Figure 3e). Statistically significant higher levels were found among the spleen when compared to the heart (*p* = 0.0114) and the liver (*p* = 0.0114). Interestingly, NHP *gpr55* was partially detected in the kidney (2/4 rhesus), liver (1/4 rhesus), and MLN (1/2 rhesus), showing more expression than the other two endocannabinoid‐like GPCRs, and a different pattern than the rodent *gpr18* and *gpr55* (Figure 3f).

*Gpr119* was partially present in the heart, kidney, liver, spleen, and visceral fat of mice, being the model with most notable expression (Figure 3g). It was undetectable in the periphery of rats (Figure 3h), but nevertheless there were significant differences found between the expression levels of the colon versus the liver (*p* = 0.0465) and the kidney versus the liver (*p* = 0.0001). In the NHP model, it was detected partially in the spleen of 2/4 rhesus. (Figure 3i).

### 3.4 TRPV1 and TRPV2 nociception channels have limited translational applicability to humans

**FIGURE 4:**
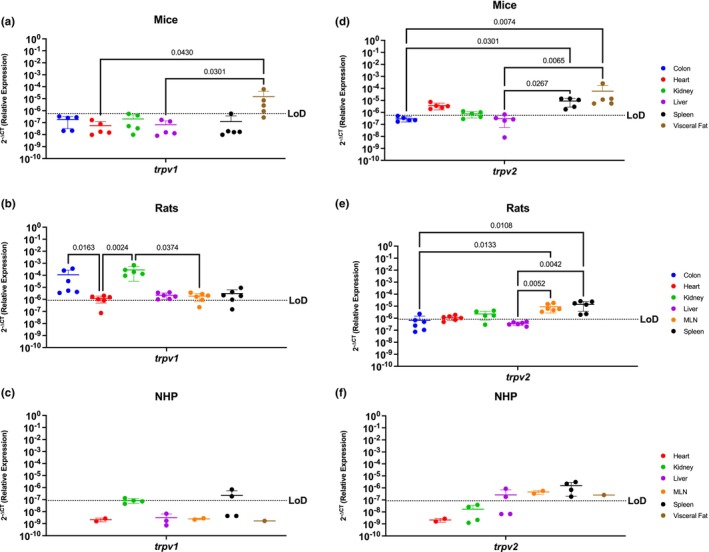
Peripheral TRPV1 and TRPV2 Nociception Channels Have Limited Translational Applicability to Humans. Relative expression of *trpv1* and *trpv2* was determined using qPCR from the colon, heart, kidney, liver, MLN, spleen, and visceral fat from mice, rats and NHP. (a) *Trpv1* was detected partially in the colon (3/5 mice), kidney (2/5 mice), and the visceral fat (3/5 mice), having significantly different expression levels among the visceral fat and the expression levels in the heart and liver. (b) *Trpv1* was detected in the colon, heart (5/6 rats), kidney, liver, MLN (5/6 rats), and spleen (5/6 rats). Significant differences were found between the kidney and the other organs, except for the colon which showed comparable expression levels. (c) *Trpv1* was detected in the kidney (1/4 rhesus) and the spleen (2/4 rhesus). (d) *Trpv2* mRNA was detected in the heart, kidney (4/5 mice), liver (1/6 mice), spleen, and visceral fat, having higher significant levels in the last two. (e) *Trpv2* was detected in the colon (1/6 rats), heart (4/6 rats), kidney (4/5 rats), MLN, and spleen. T*rpv2* was similar between immune organs, and they are both significantly different when compared with the metabolic organs. (f) *Trpv2* showed broader detection, being detected in the liver (2/4 rhesus), MLN, spleen, and visceral fat. Data are graphed as the mean ± SD.

Nociception channels are widely studied in their response to painful stimuli (Bevan et al., 2014; Kojima & Nagasawa, 2014). *Trpv1* mRNA was detected in the colon, kidney, and visceral fat of mice, having significant differences when comparing the visceral fat with the heart (*p* = 0.0430) and liver (*p* = 0.0301) (Figure 4a). In contrast, rats had a wider expression of this gene, which was detected in all evaluated organs (Figure 4b). *Trpv1* was highest in the rat colon and kidney, being statistically increased as compared to the heart (*p* = 0.0163 for colon vs. heart and *p* = 0.0024 for kidney vs. heart), and between the kidney vs. MLN (*p* = 0.0374). NHP were comparable to mice and only had detectable *trpv1* in the kidney and spleen (Figure 4c).

*Trpv2* mRNA was widely present in mouse with expression in all the organs studied (Figure 4d). Even still, *trpv2* was not expressed equally but instead was significantly higher in the spleen and the visceral fat as compared to colon (*p* = 0.0301 and *p* = 0.0074) and liver (*p* = 0.0.0267 and *p* = 0.00065). *Trpv2* was detected throughout all the organs in the rat, except for the liver (Figure 4e). Of the organs in which it was expressed, *trpv2* was most highly present in the rat MLN and spleen, reaching statistical significance with the colon (*p* = 0.0133 and *p* = 0.0108, respectively) and the liver (*p* = 0.0052 and *p* = 0.0042, respectively). Interestingly, the NHP model was the only preclinical model where *trpv2* was present in liver (Figure 4f). In contrast, *trpv2* was detectable in the NHP MLN, spleen, and visceral fat, and was comparably expressed across organs without any significant differences between them.

### 3.5 Endocannabinoid metabolic enzymes are ubiquitous in rodents, but more restricted in NHP

**FIGURE 5:**
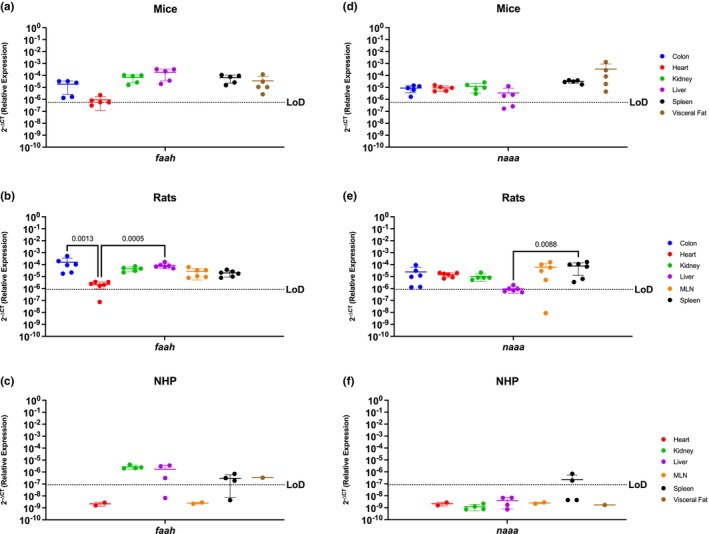
Endocannabinoid Metabolic Enzymes Are Ubiquitous In Rodents, But More Restricted In The NHP Model. Relative expression of *faah* and *naaa* was determined using qPCR from the colon, heart, kidney, liver, MLN, spleen, and visceral fat from mice, rats, and NHP. (a) F*aah* mRNA was detected in all organs tested, having differential expression between them. The heart of mice had the lowest levels for this gene. (b) *Faah* mRNA was detected in all organs included in this study. The colon of rats showed higher levels of *faah*, particularly significant when compared to expression in the heart. (c) Levels of *faah* in the NHP model were detected in the kidney, liver (3/4 rhesus), spleen (3/4 rhesus), and visceral fat. (d) N*aaa* mRNA was detected in all organs but it had partial expression and significantly lower expression in the liver (3/5 mice) when compared to the levels of expression in the spleen and visceral fat. (e) Expression of *naaa* mRNA in rats was detected in all organs and was least abundant in liver. (f) *Naaa* mRNA was detected only partially in the spleen (2/4 rhesus). Data are graphed as the mean ± SD.

*Faah* and *naaa* are ubiquitous enzymes involved in endocannabinoid degradation (Cravatt et al., 1996; Piomelli et al., 2020; Tripathi, 2020; Tsuboi et al., 2007; Van Egmond et al., 2021). In agreement with this, *faah* was present in all organs analyzed in mice with no similar levels in these peripheral organs (Figure 5a). Similarly, *faah* was present in all rat organs, having increased expression in the colon and the liver, and decreased expression in the heart when compared to the expression levels in the heart (*p* = 0.0013 and *p* = 0.00005, respectively), following a similar pattern as the mice (Figure 5b). Interestingly, NHP had a different *faah* expression pattern than the rodents. While widely detected in the kidney, liver (3/4 rhesus), spleen (3/4 rhesus), and visceral fat, *faah* was not detected in the NHP heart nor MLN (Figure 5c).

*Naaa* showed similar trends as *faah* where rodents had ubiquitous expression while NHP had more restricted expression. *Naaa* expression in mice followed a similar trend than *faah*, being detected in all peripheral organs included in this study. (Figure 5d). In rats, it was also detected in all organs, but with significantly lower expression in the liver when compared to the spleen (*p* = 0.0289) (Figure 5e). *Naaa* was only expressed in the NHP spleen (2/4 rhesus).

### 3.6 *Htr1a*, *Adora2a*, and *Adgrf1* are poorly conserved among mice, rats, and NHP

**FIGURE 6:**
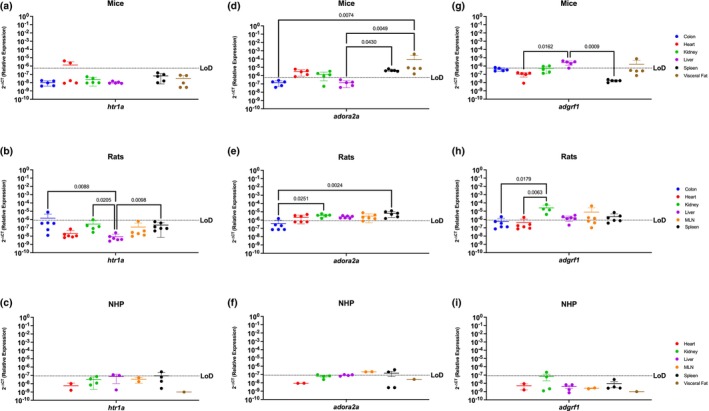
*Htr1a*, *Adora2a*, and *Adgrf1* are Poorly Conserved Among Mice, Rats, and NHP. Relative expression of *5‐htr1a*, *adora2a*, and *adgrf1* was determined using qPCR from the colon, heart, kidney, liver, MLN, spleen, and visceral fat from mice, rats, and NHP. (a) *Htr1a* was only detected partially in the colon (3/5), heart (2/5), and in the visceral fat of mice (4/5 mice) at comparable levels. (b) In rats, *htr1a* was only detected in one sample of the colon. (c) H*tr1a* in the NHP model was detected in the spleen (2/4 rhesus (d)) In mice, *Adora2a* was detected in the heart, kidney (4/5), spleen, and visceral fat. (e) *Adora2a* was detected in all organs screened, with significantly higher levels in the spleen when compared to other organs. (f) A*dora2a* in NHP was detected in the MLN and spleen. (g) *Adgrf1* was detected in the colon, kidney (3/5), and liver, having significant difference between the liver and the heart and the liver and the spleen. (h) *Adgrf1* was detected in the kidney with significant higher levels when compared to the colon, liver, and spleen. This gene was also detected in the heart (1/6 rats) and MLN (3/5 rats) (i) *Adgrf1* was detected partially in the kidney (1/4 rhesus) and the spleen (2/4 rhesus). Data are graphed as the mean ± SD.

*Htr1a* is a serotonin receptor primarily studied in the brain, while *adora2* and *adgrf1* are more widely present and implicated in inflammation, cardiovascular diseases, and cancer (Borea et al., 2018; Huang et al., 2020; Lucki, 1998; Marzo, 2018; Park et al., 2019; Pasquini et al., 2021; Saini et al., 2022). In general, *htr1a* mRNA was minimally expressed in the periphery of all three preclinical models. *Htr1a* was only detected in the heart of mice (2/5 mice), colon of rats (1/5 rats), having higher expression levels than the liver (*p* = 0.0088), and liver (2/3 rhesus) and spleen (2/4 rhesus) of the NHP preclinical model (Figure 6a–c). *Adora2a* is present to a greater extent in the periphery and was detected in all the organs tested for mice (Figure 6d) and having higher expression levels in the spleen and visceral fat when compared to the liver (*p* = 0.0430 and *p* = 0.0049, respectively) and between the spleen and the colon (*p* = 0.0074). Rats also widely expressed *adora2a* which was found in all examined organs but was lowly expressed in the colon when compared to the kidney (*p* = 0.00251) and the spleen (*p* = 0.0024) (Figure 6e). NHP also expressed *adora2a*; however, it was present only in secondary immune organs, such as the spleen and MLN (Figure 6f). *Adgrf1* was detected in the colon, kidney, and liver from mice, though it was mostly highly expressed in the liver when compared to the heart (*p* = 0.0162) and spleen (*p* = 0.0009) (Figure 6g). Rats had widespread *adgrf1* across all organs in rats, with preferential expression in the kidney when compared with the colon (*p* = 0.0179), and heart (*p* = 0.0063) (Figure 6h). *Adgrf1* had the most limited expression in NHP where it was detected only in the kidney (1/4 rhesus) (Figure 6i).

## 4 DISCUSSION

We report a comparison of the relative expression of 14 genes from the canonical and extended ECS in seven peripheral organs from three animal species and strains widely used in research, including in cannabis and cannabinoid research: C57BL/6 mice, Sprague–Dawley rats, and Rhesus macaque NHP. We identified key differences in the relative expression patterns of these evolutionary conserved, polyfunctional receptors, and found that these preclinical model systems were more dissimilar than has been previously appreciated. Indeed, there was conserved expression of only one receptor among the two rodent models (*gpr55*) and two conserved receptors between rats and NHP. Of note, *gpr55* was conserved in the colon and spleen of rodents, and among all the organs shared between rats and NHP for *ppara* and *pparg* expression. This indicates that while the ECS is highly conserved, each of the three animal species included has a differing pattern for receptor composition in their peripheral organs. This is important and has substantial implications for translation to humans and for comparison across research groups. The impact of route of administration, diet, formulation, dose, fasting versus fed state, biological sex, and metabolite distribution and bioavailability (Sharkey & Wiley, 2016) is already implicated in contributing to discrepant findings in the cannabinoid field. We propose that the unique receptor composition patterns of the preclinical model must also be considered to enhance scientific rigor and reproducibility. Indeed, as multiple canonical and extended ECS receptors are simultaneously present within a tissue, the potential for off‐target and polypharmacy effects (Almeida et al., 2013; Assareh et al., 2020; Austrich‐Olivares et al., 2022; ElBatsh et al., 2012; Fried & Nieman, 1973; García‐Gutiérrez et al., 2020; Guimarães et al., 1990; Long et al., 2010; Manwell, Charchoglyan, et al., 2014; Manwell, Ford, et al., 2014; Moreira et al., 2006; Naef et al., 2004; Niyuhire et al., 2007; Onaivi et al., 1990; Resstel et al., 2006; Sales et al., 2019; Schiavon et al., 2016; Shbiro et al., 2019; Shoval et al., 2016; Silveira Filho & Tufik, 1981; Soethoudt et al., 2017; Wilson et al., 2002; Zanelati et al., 2010; Zuardi & Karniol, 1983) is staggering as each receptor has its own unique function and signaling processes. Therefore, it is important to understand the nuances of endocannabinoid receptor tissue localization in the most common preclinical animal models.

Our findings also bring attention to the importance of additional receptors that have been understudied thus far. While the canonical ECS receptors, CB1 and CB2, have been most widely studied, our work demonstrates that the extended ECS receptor distribution represents an additional level of complexity that must be considered when performing cannabinoid studies. Indeed, these receptors and/or metabolic enzymes are simultaneously present in peripheral tissues in tandem with CB1 and/or CB2 and are also capable of mediating physiologic effects upon interacting with endo‐ and phytocannabinoids. These interactions should not be ignored as they result in a complex network of physiological pathways having diverse effects in biologic systems in the chosen preclinical model. Our results are summarized in Tables 4, 5, 6.

**TABLE 4:** Summary of the findings of the expression of the canonical and extended ECS in mice (*Mus musculus*).

Mice	Colon	Heart	Kidney	Liver	MLN	Spleen	Visceral fat
*cnr1*	+	−	+	+		+	+
*cnr2*	−	+	−	−		+	+
*ppara*	+	+	+	+		−	+
*pparg*	+	+	+	+		−	+
*gpr18*	+	−	−	−		+	+
*gpr55*	+	−	−	−		+	−
*gpr119*	+	−	+	+		+	+
*trpv1*	+	−	+	−		−	+
*trpv2*	+	+	+	+		+	+
*faah*	+	+	+	+		+	+
*naaa*	+	+	+	+		+	+
*htr1a*	−	+	−	−		−	+
*adora2A*	+	+	+	+		+	+
*adgrf1*	+	−	+	+		−	−

**TABLE 5:** Summary of the findings of the expression of the canonical and extended ECS in rats (*Ratus norvegicus*).

Rat	Colon	Heart	Kidney	Liver	MLN	Spleen	Visceral fat
*cnr1*	+	+	+	−	+	+	
*cnr2*	+	+	−	−	+	+	
*ppara*	+	+	+	+	+	+	
*pparg*	+	+	+	+	+	+	
*gpr18*	+	+	−	+	+	+	
*gpr55*	+	−	−	−	+	+	
*gpr119*	−	−	−	−	−	−	
*trpv1*	+	+	+	+	+	+	
*trpv2*	+	+	+	−	+	+	
*faah*	+	+	+	+	+	+	
*naaa*	+	+	+	+	+	+	
*htr1a*	+	−	−	−	−	−	
*adora2A*	+	+	+	+	+	+	
*adgrf1*	+	+	+	+	+	+	

**TABLE 6:** Summary of the findings of the expression of the canonical and extended ECS in Rhesus macaques (*Macaca mulatta*).

NHP	Colon	Heart	Kidney	Liver	MLN	Spleen	Visceral fat
*cnr1*		−	−	−	−	+	+
*cnr2*		−	−	+	+	+	−
*ppara*		+	+	+	+	+	+
*pparg*		+	+	+	+	+	+
*gpr18*		−	−	−	−	+	−
*gpr55*		−	+	+	+	+	−
*gpr119*		−	−	−	−	+	−
*trpv1*		−	−	+	+	−	−
*trpv2*		−	−	+	+	+	+
*faah*		−	+	+	−	+	+
*naaa*		−	−	−	−	+	−
*htr1a*		−	+	+	−	+	−
*adora2A*		−	+	+	+	+	−
*adgrf1*		−	+	−	−	−	−

### 4.1 Both canonical ECS receptors are expressed in the spleen of mice, rats and NHP

The CB1R (*cnr1* gene) is most widely studied in the brain where it mediates antinociceptive effects, appetite regulation, and interacts with phytocannabinoids (Mackie, 2005; Pertwee, 1997). However, CB1R is also present in multiple peripheral sites, including fat, lungs, and reproductive organs where its plays roles in regulating inflammation and obesity (Mackie, 2005; NCBI, 2023a). In contrast, CB2R is present primarily in peripheral organs, such as the spleen and MLN, and is most widely implicated in immune cell functions (NCBI, 2023b).

Here, we report that *cnr1* and *cnr2* mRNA were detected in peripheral organs of all three preclinical animal models, in agreement with existing studies. However, we expand on this knowledge to identify similarities and key differences among the model systems. *Cnr1* was most highly present in the colon and the visceral fat tissue for mice, while in rats its highest levels instead occurred in the kidney and colon. The NHP model had more limited *cnr1* where it was detectable only in the spleen and visceral fat. Similar findings occurred with *cnr2*. While *cnr2* was commonly detected in the MLN and spleen of all animal models, as expected, nuanced expression also existed where it was present in the rat colon, but not in the mouse. These findings denote key differences in *cnr1* mRNA not only between rodent and NHP models, but also between mice and rats.

Importantly, we determined that both canonical receptors were detected in the spleen of all three preclinical models, suggesting that it is a well conserved candidate to study the implications of CB1 and CB2 in health and disease. However, it must be acknowledged that we also identified important differences between mice, rats, and NHP. Indeed, we identified there was a striking absence of *cnr1* mRNA in the liver of rats and NHP and of *cnr2* in the kidney of all three preclinical animal models, which is inconsistent with human expression patterns (Liu et al., 2009; Mackie, 2005, 2008). This demonstrates an important limitation in translation across species. Further, this demonstrates the necessity for comparative ECS analyses to identify appropriate preclinical animal models to determine those that are best reflective of what occurs in humans.

### 4.2 Nuclear transcription factors, *Ppara* and *Pparg*, are well conserved in all organs of mice, rats and NHP

PPARs are a group of ligand‐activated nuclear hormone receptors (*ppara*, *pparb/d*, and *pparg*) that interact with Retinoid X Receptor to act as transcription factors that regulate gene expression of genes involved in energy metabolism, glucose and fat metabolism, and inflammation (Grygiel‐Górniak, 2014; Montaigne et al., 2021; Rigamonti et al., 2008; Tyagi et al., 2011). In humans, mice, and rats, *ppara* is ubiquitous, but in rodents it has biased expression in high energy requiring organs, including the heart, liver, and kidney (NCBI, 2023c; NCBI, 2023d; NCBI, 2023e; NCBI, 2023f; NCBI, 2023g; NCBI, 2023h). In contrast, PPARG is primarily present in the fat tissue in humans and mice, while rats have highest expression in the thymus (NCBI, 2023f; NCBI, 2023g; NCBI, 2023h).

Our PPARA and PPARG findings are in agreement with existing knowledge, except for their notable lack of detection in the spleen of mice. Indeed, except for this occurrence, PPARA and PPARG were the most highly conserved ECS receptor gene evaluated, having comparable detection among all organs across all preclinical models. Interestingly, *ppara* and *pparg* mRNA were detected ubiquitously in all evaluated organs in the rat and NHP model, making them possible candidates to study the peripheral expression of these receptors. These findings demonstrate high translational potential for *ppara* and *pparg* and provide implications for evaluating how cannabinoids may impact energy homeostasis (Tyagi et al., 2011), macrophage activation, insulin sensitivity, (Janani & Ranjitha Kumari, 2015; Rangwala & Lazar, 2004; Remels et al., 2009), and anti‐inflammatory pathways through NF‐kB inhibition (Remels et al., 2009; Zhang et al., 2014).

### 4.3 Endocannabinoid‐like GPRs are preferentially expressed in lymphoid organs and the visceral fat

There is limited understanding of the endocannabinoid‐like GPRs, which in humans is restricted to detection of *gpr18* and *gpr55* in lymphoid tissue and reproductive organs (NCBI, 2023i; NCBI, 2023j), and *gpr119* in the GI tract (NCBI, 2023k; NCBI, 2023l). Here, we report that, overall, the GPRs had limited expression across all preclinical models evaluated. Further, when detected, there were marked organ‐ and species‐specific differences. These GPRs were most consistently detected in the spleen in all evaluated animal models, except for the clear absence of *gpr119* in the spleen of the rat model. Additionally, in the rat and NHP model, *gpr18 and gpr55* were highly expressed, whereas in the mice model *gpr119* had the most abundance. In sum, these findings demonstrate that the rodent models represent the best preclinical model to evaluate endocannabinoid‐mediated GPR activation in vivo. Further, we identify the spleen as the most attractive therapeutic option to target the GPRs as it has the most consistent expression across all evaluated models.

While their endogenous functions are incompletely understood, the GPRs have clinically relevant implications, including *gpr18's* roles in intracellular calcium, immunomodulation, cancer, metabolism, and intraocular pressure (Bradshaw et al., 2009; Kohno et al., 2006; Morales et al., 2020; Morales & Reggio, 2017); g*pr55's* effects on bone physiology and intracellular signal transduction involving the activation of NF‐κB, NFAT, CREB, and ATF2 (Henstridge et al., 2009; Howlett et al., 2010; Lauckner et al., 2008; Leyva‐Illades & DeMorrow, 2013; Morales & Reggio, 2017; Oka et al., 2007; Yin et al., 2009); and g*r119's* involvement in glucose homeostasis and insulin secretion and sensitivity (Abdulkareem et al., 2021; Lum et al., 2010; McPartland et al., 2014; Prömel et al., 2012; Russo, 2016).

### 4.4 TRPV1 and TRPV2 nociception channels have limited translational applicability to humans

*Trpv1* and *trpv2* are ion channels that allow passage of essential ions (i.e., Na^2+^ and Ca^2+^) through the cell membrane (Bevan et al., 2014; Kojima & Nagasawa, 2014). These ionotropic receptors are involved in noxious stimuli such as pain, heat, and inflammation and its expression is ubiquitous in humans (Aghazadeh Tabrizi et al., 2017; Gorbunov et al., 2019). Here, we report marked differences in these receptors. Rats had the most similar *trpv1* expression patterns to humans as it was widely expressed, whereas it was more limited in mice and NHP. While *trpv2* was more abundant in all three preclinical animal models, the only organ with shared expression among all three preclinical models was the spleen. Expression in the colon, heart, and kidney was detected between rodents but not in NHP model. These discrepancies with human expression patterns are marked and demonstrate relatively poor translational potential. While these models are invaluable tools to evaluate the function of these receptors, care must be taken in drawing conclusions to the human condition. This suggests high potential for failure of preclinical endocannabinoid studies that aim to evaluate the roles of *trpv1* in hyperalgesia, body temperature control, diabetes, hormone secretion, epilepsy and hearing (Aghazadeh Tabrizi et al., 2017), as well as *trpv2* in cancer and cardiovascular dysfunction (Gorbunov et al., 2019; Khan et al., 2019; Mangal et al., 2021; Muller et al., 2019).

### 4.5 FAAH and NAAA endocannabinoid metabolic enzymes are ubiquitous in rodents, but more restricted in the NHP model

FAAH and NAAA are important components of the ECS through endocannabinoid regulation that are ubiquitously expressed in humans (Cravatt et al., 1996; Piomelli et al., 2020; Tsuboi et al., 2007). Our results identify that *faah* and *naaa* are ubiquitously expressed in the peripheral organs of rodents. While there were statistically significant differences among the organs, the mRNA for these metabolic enzymes were always detectable in mice and rats. Surprisingly, there was limited expression in the peripheral organs of the NHP model. Notably, *faah* was not present in the NHP heart and MLN, while *naaa* was undetectable in all organs except for spleen. This suggests that rodent models may have better preclinical utility to perform cannabinoid studies focused on targets of *faah* and *naaa*. This realization is important as these enzymes are essential in regulating endocannabinoid tone, which when dysregulated leads to pathology (McPartland et al., 2014; Russo, 2016). Inhibiting these endocannabinoid catabolic enzymes is of major therapeutic interest as FAAH inhibitors are suggested as therapeutic targets for a group of diseases related to endocannabinoid level deficiencies termed “Clinical Endocannabinoid Deficiency Syndrome,” which have implications for migraine, fibromyalgia, and irritable bowel syndrome (McPartland et al., 2014; Russo, 2016).

### 4.6 *Htr1a*, *Adora2a*, and *Adgrf1* are poorly conserved among mice, rats, and NHP

*Htr1a* and *adora2a* have been primarily studied in the context of the brain, while *adgrf1* is present in the kidney (Borea et al., 2018; Lucki, 1998; Lum et al., 2010; Prömel et al., 2012; Saini et al., 2022). We identified minimal conservation of *htr1a*, *adora2a*, and *adgrf1* among the three animal models. In fact, each species had only one organ where these genes were co‐expressed: visceral fat for mice, colon for rat, and kidney for NHP. This dissimilarity in expression patterns warns that caution must be taken when evaluating cannabinoid‐mediated effects on *htr1a*, *adora2a*, and *adgrf1* in efforts to identify new therapeutic targets, as the potential for limited translation is high. This is the most poignant demonstration of the care that must be taken when selecting preclinical animal models for endocannabinoid studies. The translational limitations of these receptors have clinical implications as *htr1a* has been extensively studied as a target for mood disorders, a*dora2a* is suggested as a therapeutic target for neurodegenerative disorders, blood–brain barrier integrity, immunosuppression, cancer, and angiogenesis (Borea et al., 2018; Pasquini et al., 2021), and a*dgrf1* is proposed as a novel therapeutic for cancer and inflammation (Abdulkareem et al., 2021; Huang et al., 2020; Park et al., 2019).

### 4.7 Limitations

This study has potential limitations. First, despite our effort to include both sexes in our study, female NHP were not included as they are an acutely scarce resource in accordance with the Sex as a Biological Variable policy NOT‐OD‐15‐102 at the National Institutes of Health. Nonetheless, potential sex‐dependent differences in ECS receptor expression profiles are important to study and require careful consideration in the development of potential cannabinoid‐based therapeutic approaches. Next, our study evaluated mRNA profiles of the ECS receptor system, rather than protein expression, due to known off‐target effects on ECS receptor antibodies. However, we acknowledge that mRNA is not necessarily predictive of protein expression and will be the focus of future work. Finally, while our goal was to comprehensively evaluate the ECS receptor system, we acknowledge that important genes were omitted, including monoacylglycerol lipase (MGL) that is important in hydrolysis of the endocannabinoid 2‐AG, as our focus was on therapeutic potential of receptors that interact with phytocannabinoids.

## 5 CONCLUSIONS

The endocannabinoid system is an attractive therapeutic target for many disorders where phytocannabinoids and receptor agonists/antagonists are being considered as novel treatment strategies. However, our findings demonstrate species‐ and organ‐specific effects where there is limited overlap in expression pattern among mice, rat, and rhesus macaque preclinical models. We recommend that cannabinoid studies carefully consider the preclinical model to be included with respect to animal species, strain, genetic background, and even the site of procurement as there are reported variations in the same strains of rats obtained from different vendors (Moore et al., 2021). It is our hope that our findings will demonstrate the need to consider species‐specific differences when designing preclinical studies. Further, we urge the cannabinoid field to evaluate the extended ECS receptors, in addition to the more widely studied CB1 and CB2, as they are abundantly expressed, are activated by both endo‐ and phytocannabinoids, and represent underlying mechanisms of action for these important lipid ligands.

## AUTHOR CONTRIBUTION

JJRF participated in tissue collection, RNA extraction, and cDNA synthesis, performed the experiments, determined the relative expression of most of the genes in all animal models, created all the graphs and data analysis, and wrote the first draft of the manuscript. ALE participated in the mice necropsies and determined relative expression of the NHP genes *cnr1, gpr55, trpv1, trpv2*, and participated in editing the manuscript. CFM and EMW provided the rats included in this study, participated in necropsies and experimental feedback, and edited the manuscript. CJW and ASP provided the mice included in this project, participated on necropsies, and provided experimental feedback and edited the manuscript. REW performed experiments of mice *ppara*. DWW conceived the project idea and experimental design, provided direct guidance, funding for these experiments, and edited the manuscript.

## FUNDING INFORMATION

Research reported in this publication was supported by the National Institute on Drug Abuse of the National Institutes of Health under award number R01DA052859 and U01DA058527 (DWW) and the National Institute of Neurologic Disorders and Stroke award number K00NS118713 (CJW). The authors also acknowledge mentorship to DWW and procurement of pilot funds to ALE from the Johns Hopkins University Center for AIDS Research (P30AI094189), Diversity Supplement funded by R01‐DA052859‐03S1 to JJRF. REW is a Solomon H. Snyder Fellow at the Neuroscience Training Program at Johns Hopkins University. The content is solely the responsibility of the authors and does not necessarily represent the official views of the National Institutes of Health.

## CONFLICT OF INTEREST STATEMENT

The authors declare no financial interest, conflict of interest, or any competing interest of any kind.

## Data Availability

The data gathered in this study are compiled and stored according to NIH data management, storing and sharing policies. Data will be available 1 year after the study has been published and can be accessed using the following link doi: 10.17632/t6yd6j6bm6.1.
